# Supplementary material for: Kombucha: Production and Microbiological Research
Source: Foods. 2022 Oct 31;11(21):3456. doi: 10.3390/foods11213456 (PMC9658962; doi:10.3390/foods11213456)
Supplement: Supplementary file 1 [file foods-11-03456-s001.zip › foods-1955037-supplementary.pdf]

Supplementary materials

Table S1 Commonly used media for the isolation, cultivation and differentiation of acetic acid bacteria.

Medium	Components (g/L) or (mL/L)	References
Glucose yeast extract carbonate (GYC)	Glucose (100), yeast extract (10), calcium carbonate (20), bacteriological agar (15)	[63,64,65]
Glucose yeast extract (GY)	Glucose (20), yeast extract (10), bacteriological agar (20)	[60,66]
Yeast extract Peptone Mannitol (YPM)	Yeast extract (5), mannitol (25), peptone (3), bacteriological agar (12)	[64]
Malt yeast extract (MYA)	Malt extract (15), yeast extract (5), Bacteriological agar (15)	[64,66]
Reinforced Acetic acid ethanol (RAE)	Glucose (40), yeast extract (10), peptone (10), Na_2PO_4*$2H_2O$ (3.38), citric acid x H_2O, ethanol (20), acetic acid (50), bacteriological agar (20)	[67]
Basal medium ethanol (BME)	Yeast extract (0.5), vitamin-free casamino acids (3), ethanol (3), bacteriological agar (15)	[68]
Carr	Yeast extract (30), ethanol (20), bromocresol green (0.022), bacteriological agar (20)	[69]
Glucose yeast extract acetic acid ethanol (GYAE)	Glucose (50), yeast extract (10), acetic acid (10), ethanol (20), bacteriological agar (15)	[70]
Glucose yeast extract ethanol calcium carbonate (GYEC)	Glucose (10), yeast extract (10), calcium carbonate (20), ethanol (30), bacteriological agar (15)	[71]
Glucose yeast extract peptone (GYP)	Glucose (30), yeast extract (5), peptone (2), bacteriological agar (15)	[72]
Hestrin-Schramm (HS)	Glucose (20), yeast extract (5), peptone (5), Na_2HPO_4 (2.7).	[73]
Sorbitol yeast extract peptone (SYP)	Sorbitol (50), yeast extract (5), peptone (3), bacteriological agar (15)	[60]
Yeast extract glucose mannitol (YGM)	Glucose (20), mannitol (20), yeast extract (10), acetic acid (5), ethanol (20)	[74]

Table S2. Commonly used media for the isolation, cultivation and differentiation of yeast from food products [77].

Medium	Component (g/L) or (mL/L)
Sabouraud glucose agar (SGA)	Glucose (10), Peptone (10) bacteriological agar (15)
Potato dextrose agar (PDA)	Glucose (20), potato infusion (500), bacteriological agar (15)
Malt extract agar (MEA)	Malt extract (20), peptone (5), bacteriological agar (15)
Acidified potato dextrose agar (APDA)	Glucose (20), potato infusion (500), lactic acid solution (5), bacteriological agar (15)
Acidified Tryptone glucose yeast extract agar (ATGY)	Tryptone (5), glucose (100), yeast extract (5), glacial acetic acid (10), bacteriological agar (15)
Oxytetracycline glucose yeast extract agar (OGY)	Glucose (20), agar (12), yeast extract (5), oxytetracycline solution (10)
Yeast extract glucose chloramphenicol (YGC) agar	Glucose (20), agar (14.9), yeast extract (5), Chloramphenicol (0.1)
Rose Bengal chloramphenicol agar (RBC)	Glucose (10), Papaic digest of soybean meal (5), KH_2PO_4 (1), $MgSO_4$*$7H_2O$ (0.5), Rose Bengal (0.05), chloramphenicol solution (10)
Dichloran Rose Bengal chloramphenicol agar (DRBC)	Glucose (10), peptone (5), KH_2PO_4 (1), $MgSO_4$*$7H_2O$ (0.5), Rose Bengal (5% w/v, 0.5), chloramphenicol (0.1), dichloran (0.2% w/v in ethanol, 1), bacteriological agar (15)
